# Pine Ward Quality Improvement Project

**DOI:** 10.1192/bjo.2026.11409

**Published:** 2026-06-30

**Authors:** Karim Kerolos, Alessandro Malfatto, Theodoros Mavrogiannidis, Anthony Singh

**Affiliations:** 1 Norfolk and Suffolk NHS Trust, Kings Lynn, United Kingdom; 2 CNWL, London, United Kingdom

## Abstract

**Aims:**

There has been a recent increase in incidents in Pine Ward at Park Royal mental health unit. Feedback from staff/patients is that they don't feel safe and there has been staff sickness due to physical aggression towards them. We proposed to do this QI project in order to improve the staff and patient safety on the ward.

The aim of this QI project was to improve safety for patients and staff on Pine Ward by 20% by end of December 2025, to reduce incidents of violence and aggression and strengthen communication and team work.

**Methods:**

Key changes: Weekly community meeting; Improve observation of patient positive strengths; Messages in nursing station on using soft words; and Section 17 leave flow chart given to patients.

Data collection (November–December 2025)

• We reviewed patient and staff safety on ward from mid November to end of December.

• We collected data on the following questions:

– Patient staff conflict survey.

– Patient safety feedback form.

– Staff safety feedback form.

**Results:**

Patient safety improved by 25% and staff safety improved by 30%.

Incidents of Verbal aggression in day shift decreased from 4 to 2 per shift.

Incidents of Aggression towards objects in day shift decreased from 2 to 1.

Decrease PRN psychotropic use in day shift from 4 to 2.

**Conclusion:**

Communication between patients and staff is key for safety. Community meeting is a great opportunity to explore patient ideas and can be used for education about section 17 leave.

Thinking about patient qualities and strength affect how we perceive patient as person and influence positive therapeutic relations.

Verbal aggression against staff is a key concerns and needs to be worked on. Record of incidents of aggression on datix to be reviewed in future audit.

